# Hypoxia‐Inducible Factor‐2α Signaling in the Skeletal System

**DOI:** 10.1002/jbm4.10733

**Published:** 2023-02-26

**Authors:** Sarah V Mendoza, Damian C Genetos, Clare E Yellowley

**Affiliations:** ^1^ Department of Anatomy, Physiology, and Cell Biology, School of Veterinary Medicine University of California, Davis Davis CA USA

**Keywords:** TRANSCRIPTION FACTORS, GENETIC ANIMAL MODELS, OSTEOBLAST, OSTEOCYTE, OSTEOCLAST

## Abstract

Hypoxia‐inducible factors (HIFs) are oxygen‐dependent heterodimeric transcription factors that mediate molecular responses to reductions in cellular oxygen (hypoxia). HIF signaling involves stable HIF‐β subunits and labile, oxygen‐sensitive HIF‐α subunits. Under hypoxic conditions, the HIF‐α subunit is stabilized, complexes with nucleus‐confined HIF‐β subunit, and transcriptionally regulates hypoxia‐adaptive genes. Transcriptional responses to hypoxia include altered energy metabolism, angiogenesis, erythropoiesis, and cell fate. Three isoforms of HIF‐α—HIF‐1α, HIF‐2α, and HIF‐3α—are found in diverse cell types. HIF‐1α and HIF‐2α serve as transcriptional activators, whereas HIF‐3α restricts HIF‐1α and HIF‐2α. The structure and isoform‐specific functions of HIF‐1α in mediating molecular responses to hypoxia are well established across a wide range of cell and tissue types. The contributions of HIF‐2α to hypoxic adaptation are often unconsidered if not outrightly attributed to HIF‐1α. This review establishes what is currently known about the diverse roles of HIF‐2α in mediating the hypoxic response in skeletal tissues, with specific focus on development and maintenance of skeletal fitness. © 2023 The Authors. *JBMR Plus* published by Wiley Periodicals LLC on behalf of American Society for Bone and Mineral Research.

## Introduction

Molecular oxygen (O_2_) is required for vital cellular processes that maintain cell function. O_2_ is an obligate component of mitochondrial respiration, amino acid catabolism, lipid metabolism, and other biochemical reactions essential for sustaining life.^(^ ^1^ ^)^ The bioavailability of molecular O_2_ is dynamic, driven by blood flow and oxygen saturation.^(^ ^2^ ^)^ Pathophysiological insults such as vascular disease, heart failure, or fracture disrupt tissue perfusion, locally depriving cells and tissues of oxygen (hypoxia). Adaptation to O_2_ bioavailability occurs predominately via transcriptional changes that shift metabolism to anaerobic glycolysis, increase glucose uptake from the pericellular environment, and reestablish tissue vascularization to restore cellular and tissue function.^(^ ^3^ ^)^

Oxygen‐sensing transcription factors termed hypoxia‐inducible factors (HIFs) drive many, but not all, adaptive responses to hypoxia. HIFs are heterodimeric transcription factors composed of an α subunit and β subunit.^(^ ^4^ ^)^ The α subunit is constitutively expressed in the cytosol, where its stability is determined by cellular O_2_ levels, whereas the β subunit (HIF‐β, or aryl hydrocarbon receptor nuclear translocator [ARNT]) is constitutively expressed in the nucleus and is stabilized independent of cellular O_2_.^(^ ^5^ ^)^ Constitutively expressed HIF‐β subunits bind to one of three HIF‐α isoforms (HIF‐1α, HIF‐2α, and HIF‐3α), recruit the transcriptional coactivator p300 and/or CREB‐binding protein (CBP), and bind to hypoxia‐response elements (HRE) in responsive genes to initiate gene transcription.^(^ ^6^ ^)^ HIF‐1α and HIF‐2α are both principal transcriptional regulators, whereas HIF‐3α antagonizes HIF‐1α‐ and HIF‐2α‐ mediated transcription.^(^ ^7^, ^8^ ^)^ HIF‐1α and HIF‐2α bind HIF‐ß isoforms ARNT (HIF‐1ß) or ARNT2.^(^ ^9^, ^10^, ^11^ ^)^ HIF‐α isoforms share a 48% overall amino acid sequence identity,^(^ ^12^ ^)^ whereas HIF‐ß isoforms share 63% identity.^(^ ^13^ ^)^

## PHD/VHL Regulation of HIF Signaling

**Fig. 1:**
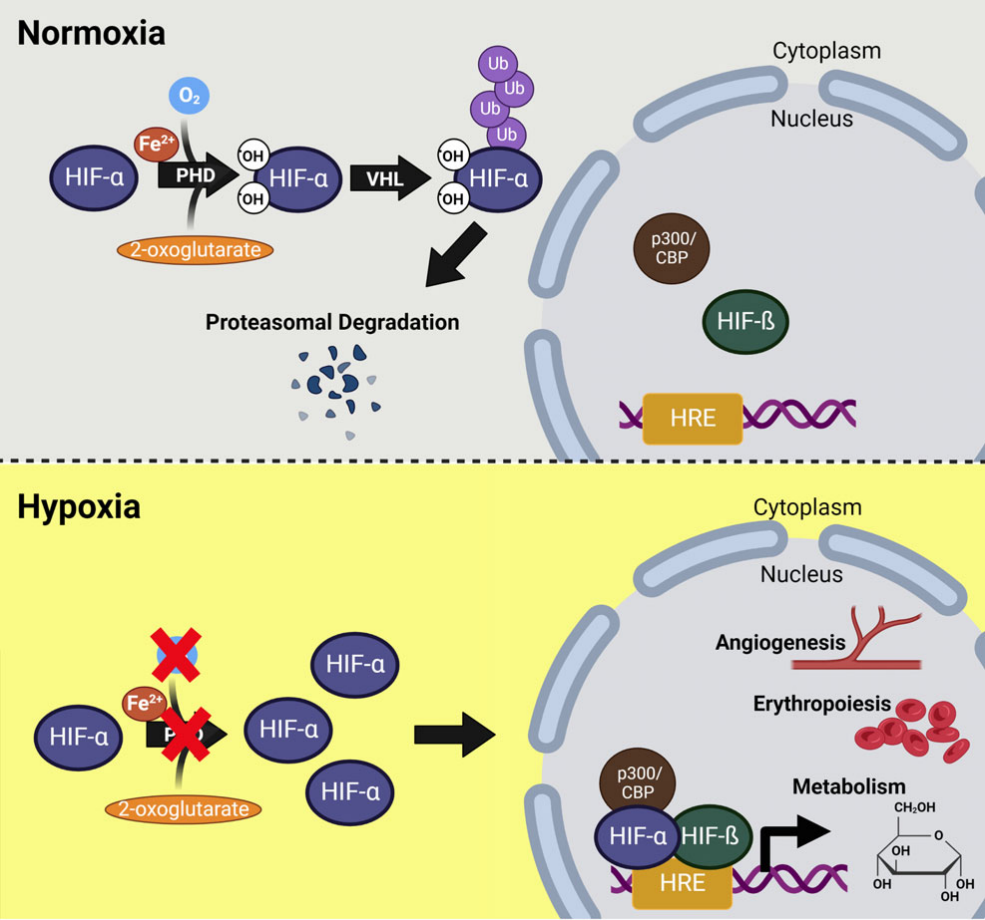
Hypoxia‐inducible factor (HIF) signaling pathway. Under normoxia, prolyl hydroxylases (PHDs) hydroxylate hypoxia‐inducible factor‐α subunits (HIF‐α) on two proline residues, triggering Von Hippel‐Lindau (VHL)‐mediated poly‐ubiquitination and proteasomal degradation. The cofactors α‐ketoglutarate and ferrous iron (Fe^2+^) are required for the hydroxylation reaction. Under hypoxia, hydroxylation is inhibited by the lack of oxygen and HIF‐α dimerizes with constitutively expressed nucleus‐confined HIF‐β, recruits CREB‐binding protein (CBP) and p300, creating a transcriptionally active HIF complex that binds to hypoxia‐responsive elements (HRE) on DNA, then induces hypoxia‐inducible genes involved in angiogenesis, erythropoiesis, and metabolism.

Under conditions of sufficient molecular O_2_ (normoxia), prolyl hydroxylase domain (PHD) proteins use O_2_ as an obligate cofactor to hydroxylate HIF‐α subunits to promote their degradation. Hydroxylated HIF‐α subunits are recognized and polyubiquitinated by Von Hippel‐Lindau (VHL) tumor suppressor protein, which is the recognition component of an E3 ubiquitin‐protein ligase, and are targeted for 26S proteasomal degradation^(^ ^14^ ^)^ (Fig. 1, top). Under hypoxic conditions, reduced O_2_ availability limits HIF‐α hydroxylation by PHD proteins, thereby preventing recognition by VHL and HIF‐α degradation. Stabilized HIF‐α subunits then accumulate in the cytosol, translocate to the nucleus, and dimerize with nuclear HIF‐ß subunits (Fig. 1, bottom).

**Fig. 2:**
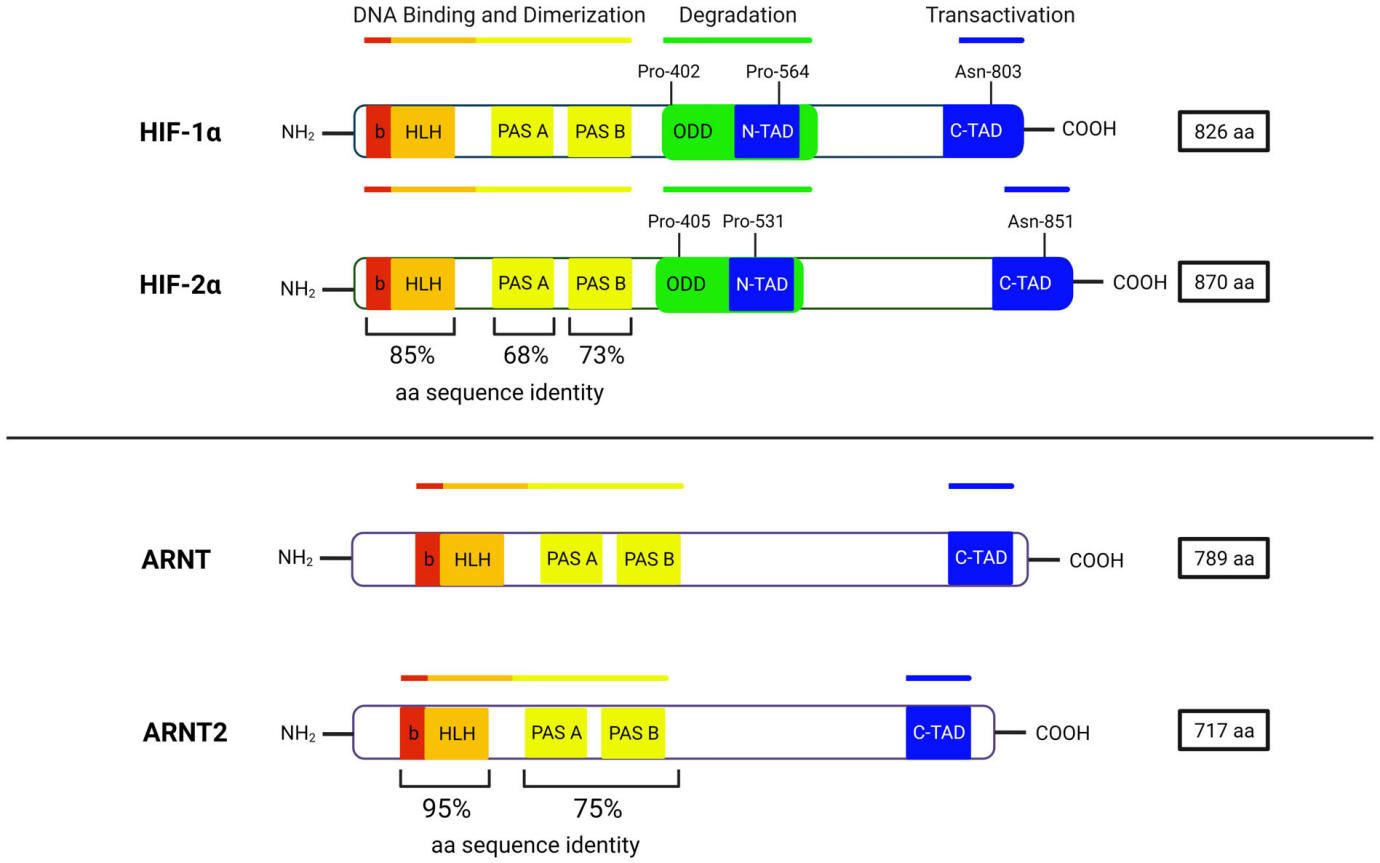
Hypoxia‐inducible factor‐α (HIF‐α) and hypoxia‐inducible factor‐ß (HIF‐ß) functional domain comparison. HIF‐α isoforms are composed of a basic (b) and helix‐loop‐helix (HLH) domain, Per‐Arnt‐Sim (PAS A and PAS B) domain, an N‐terminal transactivation domain (N‐TAD), and a C‐terminal transactivation domain (C‐TAD) (top). HIF‐1α and HIF‐2α share high amino acid sequence identity in the bHLH and PAS domains but have distinct N‐TADs (top). HIF‐ß isoforms (ARNT and ARNT2) also share high amino acid sequence identity in their bHLH and PAS domains (bottom) but lack an N‐TAD region. Details explained in the text.

HIF‐α isoforms contain multiple highly conserved and functionally distinct domains. An N‐terminal basic helix‐loop‐helix (bHLH) domain aids in both binding to HRE sequences and dimerizing with HIF‐ß.^(^ ^15^ ^)^ The bHLH domain is followed by a Per‐Arnt‐Sim (PAS) domain that predominantly contributes to dimerization with HIF‐ß.^(^ ^15^ ^)^ HIF‐α/β dimerization occurs via interactions between bHLH‐PAS domains on both HIF‐α and HIF‐ß subunits.^(^ ^15^, ^16^, ^17^, ^18^ ^)^ The PAS domain on HIF‐ß subunits is composed of a PAS‐A region that dimerizes with the bHLH‐PAS region on HIF‐α subunits and a PAS‐B region that undergoes energetic and conformational changes in response to exogenous and endogenous signals, regulating protein activity.^(^ ^19^ ^)^ The highest shared amino acid sequence identity of HIF‐α isoforms occurs in bHLH (85%), PAS‐A (68%), and PAS‐B (73%) domains,^(^ ^12^ ^)^ whereas HIF‐ß isoforms share 95% identity in the bHLH domain and 75% in the PAS domain^(^ ^13^ ^)^ (Fig. 2). Heterodimeric HIFs then bind HRE in transcriptional regulatory elements, where CREB‐binding protein (CBP) and/or p300 is recruited to facilitate expression of hypoxia‐responsive genes.^(^ ^6^ ^)^

Oxygen‐dependent domains (ODDs) on HIF‐α subunits contain regulatory hydroxylation sites for PHD proteins. PHD hydroxylation occurs at proline residues at Pro‐402 and Pro‐564 in HIF‐1α^(^ ^20^ ^)^ and Pro‐405 and Pro‐531 in HIF‐2α,^(^ ^21^ ^)^ a process catalyzed by ferrous iron (Fe^2+^) and 2‐oxoglutarate^(^ ^22^ ^)^ (Fig. 2). HIF‐2α is hydroxylated less efficiently by PHD proteins, resulting in stabilization at higher O_2_ tensions compared with HIF‐1α.^(^ ^23^ ^)^ The shift from HIF‐1α‐ to HIF‐2α‐dependent response to chronic hypoxia occurs via microRNAs and E3 ubiquitin ligases called hypoxia‐associated factors (HAFs) that bind the ODD of HIF‐1α and induce its ubiquitination and degradation.^(^ ^24^ ^)^ In contrast, HAF binds to the region between the N‐TAD and C‐TAD on HIF‐2α and increases its activation.^(^ ^25^ ^)^ HAF expression decreases after acute hypoxia and increases during chronic hypoxia, contributing to the switch from HIF‐1α to HIF‐2α transcriptional activity.^(^ ^25^ ^)^ Thus, HIF‐1α mediates acute and HIF‐2α mediates chronic response to hypoxia.

The remaining functional domains of HIF‐α are the two transactivation domains (TADs), one located at the N‐terminus (N‐TAD) and the other at the C‐terminus (C‐TAD)^(^ ^26^ ^)^ (Fig. 2). HIF‐1α and HIF‐2α isoforms have structural and functional similarity in DNA binding/dimerization domains at their N termini and C‐TADs at their C‐termini, while their N‐TADs are distinct.^(^ ^26^ ^)^ Structural differences in the N‐TAD of HIF‐1α or HIF‐2α impart target gene specificity, as replacement of HIF‐2α N‐TAD with HIF‐1α N‐TAD is sufficient to confer HIF‐1α‐specific functionality to HIF‐2α.^(^ ^26^ ^)^ HIF‐1 DNA binding sites are closer to gene promoters, whereas HIF‐2 binds to distal enhancers, suggesting that they do not compete for binding sites and behave independently.^(^ ^27^ ^)^

HIF transcriptional activity can be suppressed by factor‐inhibiting hypoxia‐inducible factors (FIH), oxygen‐dependent hydroxylase enzymes distinct from PHDs that catalyze β‐hydroxylation of an asparagine residue (Asn‐803 in HIF‐1α and Asn‐851 in HIF‐2α) in the C‐TAD of both HIF‐α subunits, blocking coactivator association and inhibiting transcription.^(^ ^18^, ^28^, ^29^ ^)^ FIH finely modifies HIF‐α degradation by binding the C‐TAD during severe hypoxia, independently of PHD's binding at the N‐TAD that occurs during intermediate hypoxia.^(^ ^30^ ^)^ The role of PHD and FIH's hydroxylase function in mediating the adaptive hypoxia response are not redundant but synergistic, as siRNA depletion of both PHD2 and FIH are required to generate a full hypoxia response in normoxic HeLa cells.^(^ ^30^ ^)^

## Refinement of HIF Signaling via Parallel Stress Response Pathways

Despite the nature of its discovery, HIF stabilization does not occur exclusively in PHD‐dependent manners. PHD‐dependent regulation is the predominant driver of HIF‐α stabilization; however, HIF‐α signaling is also modulated by epigenetic modifications that modify *Hif1a* and *Hif2a* transcript levels. For example, Sirtuin 1 (SIRT1) is a redox‐sensing deactetylase that monitors the cell's metabolic state and mediates the cell's transcriptional response to stress by altering substrate activity, including HIF‐2α.^(^ ^31^ ^)^ Under hypoxia, SIRT1 is activated to deacetylate HIF‐2α, but not HIF‐1α, at select C‐terminal lysine residues and enhance HIF‐2α transcriptional activity. Inverse relationships between SIRT1 and HIF‐2α activity investigated via erythropoietin (*Epo*) expression changes are reported in Hep3B and HEK293 cell models^(^ ^31^ ^)^ and in a murine model of *Sirt1* ^*+/‐*^ heterozygosity.^(^ ^31^, ^32^ ^)^ Thus, SIRT1 enhances HIF‐2 signaling during hypoxia via PHD‐independent posttranslational modifications.

Modification of HIF‐α signaling can occur via the tricarboxylic acid (TCA) cycle intermediate α‐ketoglutarate, an intracellular indicator of amino acid availability and obligate substrate for PHD activity.^(^ ^33^, ^34^ ^)^ Amino acid availability is monitored by mammalian target of rapamycin complex 1 (mTORC1) that regulates protein, lipid, and nucleotide synthesis for cell growth.^(^ ^34^ ^)^ Under conditions of amino acid sufficiency, increased α‐ketoglutarate levels activate PHDs, whose activity is required for the activation of mTORC1.^(^ ^34^ ^)^ In human bone osteosarcoma epithelial cells (U2OS), α‐ketoglutarate depletion caused by amino acid starvation inactivates PHDs, reducing mTORC1 activation and preventing HIF‐1α translation.^(^ ^34^, ^35^ ^)^ In human prostate cancer cells (PC‐3), pretreatment with rapamycin strongly reduced expression of HIF‐1α target genes (*Vegf*) under hypoxia.^(^ ^35^ ^)^ Treatment of PC‐3 cells with LY294002, an inhibitor of the mTORC1 activator phosphatidylinositol‐3‐kinase (PI3K), significantly reduced *Hif1a* expression and HIF‐1α stabilization.^(^ ^35^ ^)^ PHD inactivation resulting from amino acid starvation reduces HIF‐1α expression and stabilization via the dual‐functioning oxygen and nutrient sensor α‐ketoglutarate; however, its effect on HIF‐2α is currently unknown.

Oxidative stress coordinates HIF‐α activity.^(^ ^36^ ^)^ Under hypoxia, nutrient deprivation and signaling impairment causes oxidative stress that impairs protein folding in the endoplasmic reticulum (ER); impaired protein folding triggers the unfolded protein response (UPR) to reduce protein synthesis and accumulation of unfolded proteins.^(^ ^37^, ^38^ ^)^ Protein kinase RNA‐like ER kinase (PERK) is a UPR sensor that phosphorylates eIF3e, a translation initiation factor, to inhibit translation initiation and prevent further misfolded protein generation.^(^ ^39^ ^)^ In addition to its role as a translational regulator, eIF3e was recently identified as a HIF‐2α posttranslational regulator,^(^ ^40^ ^)^ as eIF3e binds to HIF‐2α transcript and induces proteasomal degradation of HIF‐2α, independent of hypoxia.^(^ ^18^ ^)^ In vitro studies in yeast and HeLa cells revealed that eIF3e knockdown increased HIF‐2α stabilization and transcriptional activation of target genes independent of hypoxia.^(^ ^18^, ^40^ ^)^ eIF3e‐mediated HIF‐2α degradation occurs independently of VHL, as adenovirus‐induced overexpression of eIF3e and HIF‐2α in cells from the renal cell carcinoma cell 786‐O line (endogenous *Vhl*‐deficient) induced degradation of both transfected and endogenous HIF‐2α under normoxia.^(^ ^18^ ^)^ In contrast, Pereira and colleagues suggested that the UPR enhances HIF‐1α by potentiating its phosphorylation.^(^ ^41^ ^)^

Hypoxic stress also induces the activity of heat shock protein 90 (Hsp90), a chaperone protein that assists in the stabilization, folding, and assembly of many proteins, like HIF‐1α.^(^ ^42^ ^)^ Under normoxic conditions, Hsp90 binds to the PAS domain on HIF‐1α in the cytosol, inhibiting its activity concomitantly with parallel VHL‐mediated proteasomal degradation. Under hypoxia, Hsp90 binds to HIF‐1α, then translocates to the nucleus where HIF‐ß displaces Hsp90.^(^ ^42^ ^)^ Hsp90 also competes with receptor of activated protein kinase C1 (RACK1), a protein that links HIF‐1α to elongin‐C, a ubiquitin ligase similar to VHL, destabilizing it and promoting its polyubiquitinization and proteasomal degradation.^(^ ^43^ ^)^ Thus, posttranslational regulation of HIF‐2α can occur independently of oxygen availability.

HIF‐1 and HIF‐2 play shared and independent roles in adaptive responses to hypoxia, yet precise mechanisms and functional effects of HIF‐1 and HIF‐2 require clarification. Current HIF pathway‐related studies predominantly focus on HIF‐1;^(^ ^7^, ^44^, ^45^ ^)^ less is known about HIF‐2 in development and disease.

## HIF Isoform Function

HIF‐1 and HIF‐2 are highly homologous. Unsurprisingly, they induce the transcription of common targets like erythropoietin (*Epo*) for erythrocyte proliferation, vascular endothelial growth factor (*Vegf*) for angiogenesis, delta‐like 4 (*Dll4*) for vascular formation, migration, and invasion, and glucose transporter‐1 (*Glut1*) to increase pericellular glucose uptake; yet HIF‐1α and HIF‐2α induce transcription of unique targets as well.^(^ ^3^, ^46^, ^47^ ^)^ HIF‐1α's principal role is to shift metabolism from oxidative to glycolytic metabolism by activating expression of key glycolytic regulatory enzymes like hexokinase 2 (*Hk2*), phosphofructokinase (*Pfkm*), and pyruvate kinase M (*Pkm*).^(^ ^48^ ^)^ HIF‐1α also activates expression of pH regulatory proteins like carbonic anhydrase IX (*Ca9*) that equilibrate reactions between carbon dioxide, bicarbonate, and protons (H^+^) for adaptation to acidosis.^(^ ^48^, ^49^ ^)^ In contrast, HIF‐2α induces expression of angiopoietin 2 (*Ang2*) and VEGF receptor tyrosine kinase 2 (*Flk1*) for angiogenesis, and divalent metal transporter 1 (*Dmt1*), cytochrome b reductase 1 (*DcytB*) and the iron exporter ferroportin (*Fpn*) for iron homeostasis.^(^ ^50^, ^51^ ^)^ Thus, HIF‐1α plays a predominant role in metabolic adaptation to hypoxia, whereas HIF‐2α predominantly regulates the angiogenic and iron homeostatic response.

The hypoxic response is composed of a carefully orchestrated shift from HIF‐1α activity during acute hypoxia to HIF‐2α during chronic hypoxia, termed the HIF switch.^(^ ^52^, ^53^ ^)^ In human umbilical vein endothelial cells (HUVECs) exposed to hypoxia, HIF‐1α rapidly accumulates during acute hypoxia (4 hours), reduces during the acute‐chronic hypoxia transition (8 hours), and drastically reduces thereafter during chronic hypoxia exposure (24 hours).^(^ ^54^, ^55^ ^)^ HIF‐2α levels reach a peak during the acute‐chronic hypoxia transition (8 hours) and are sustained during chronic hypoxia exposure (24 hours).^(^ ^54^, ^55^ ^)^ *Hif1a* mRNA levels were reduced post‐HIF‐1α peak (at 8 hours), whereas *Hif2a* mRNA levels were sustained at higher levels throughout the time course.^(^ ^54^ ^)^ In this study, *Hif2a* mRNA levels were twofold higher than *Hif1a* under normoxia and were fivefold higher during chronic hypoxia (24 hours).^(^ ^54^ ^)^ Time‐course mRNA studies on human endothelial cells under hypoxia revealed that differences in *Hif1a* and *Hif2a* mRNA levels were the result of microRNA destabilization of the adenylate‐uridylate‐rich element (ARE) at the 3' end of *Hif1a* and *Hif2a* transcript untranslated region. The *Hif1a* 3' UTR is highly sensitive to ARE‐dependent destabilization compared with the 3' UTR of *Hif2a*.^(^ ^55^, ^56^ ^)^ Thus, miRNA‐driven destabilization of *Hif1a* contributes to the initial reduction in *Hif1a* transcript in the shift from acute to chronic hypoxia, although this is insufficient to drive the HIF switch. Other factors contribute to the HIF switch such as SIRT1, which inhibits HIF‐1α activity by preventing p300/CBP binding but promotes HIF‐2α transactivation and transcriptional activity.^(^ ^31^ ^)^ HAFs contribute to the HIF switch by inhibiting HIF‐1α by inducing its proteasomal degradation and promoting HIF‐2α activity by promoting its transactivation.^(^ ^25^ ^)^

## HIF Isoform Expression

**Fig. 3:**
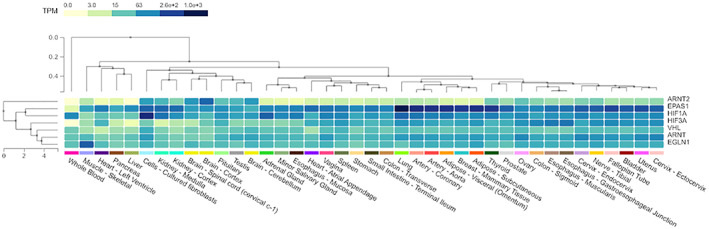
Expression patterns of regulatory and functional hypoxia‐associated proteins across tissue types. mRNA expression levels of ARNT2, EPAS1 (*Hif2a*), HIF1A, VHL, ARNT, and EGLN1 (*Phd2*) in transcripts per million (TPM) generated from the GTEx Portal: GTEx Multi‐Gene Single Cell Query. Color signifies low mRNA expression (beige) to high mRNA expression (dark blue).

HIF‐1α is ubiquitously expressed in mammalian tissues and cell types,^(^ ^57^ ^)^ whereas HIF‐2α is expressed in more vascularized tissues like heart, placenta, and kidney.^(^ ^12^, ^58^ ^)^ Figure 3 shows the mRNA distribution of HIF signaling proteins across different tissue types generated with clinical RNAseq data from healthy human adults (GTExPortal). ARNT is generally expressed at higher levels than ARNT2 across tissue types, whereas VHL and PHD2 (*Egln1*) expression are consistent across tissue types. *Hif2a* expression varies widely across tissues and suggests overall higher expression of *Hif2a* compared with *Hif1a* in these tissue types. Certainly, HIF‐α activity is predominantly regulated via posttranslational modifications, and the data described in Figure 3 show basal *Hif1a* and *Hif2a* mRNA expression levels that are susceptible to destabilization and degradation by oxygen‐dependent or independent microenvironment changes.

HIF‐α isoforms are required for normal mammalian development, as global deletion of HIF‐α isoforms causes pleiotropic developmental defects.^(^ ^59^ ^)^ *Hif1a* deletion produces severe cardiac malformation, erythropoiesis impairment, and blood vessel defects resulting in mid‐gestation lethality, indicating that the development of the circulatory system requires HIF‐1α.^(^ ^60^, ^61^ ^)^ In contrast, global deletion of *Hif2a* caused vascular defects during development, impaired lung development, and hematopoietic cell production.^(^ ^62^ ^)^ These findings emphasize the necessity of HIF isoforms in mammalian development; however, HIF‐α isoforms remain a functional requirement of a multitude of adult tissues like bone.

## HIF Signaling in the Skeleton

The following text describes the effects of HIF pathway manipulation in skeletal cells in vivo and in vitro and is summarized in Tables 1 and 2.

**Table 1:** Skeletal Effects of Hypoxia‐Inducible Pathway‐Associated Protein Genetic Deletion

Cre‐driver/Gene	*Phd*	*Hif1a*	*Hif2a*	*Vhl*
*Prx1‐cre*		Massive chondrocyte death and severe limb abnormalities^(^ ^80^ ^)^	High bone mass phenotype with increased trabecular and cortical bone without changes in osteoclast number^(^ ^80^ ^)^	Delayed bone marrow cavity development^(^ ^75^ ^)^
				Dramatic delay of chondrocyte hypertrophy, impairment of chondrocyte differentiation, and massive postnatal chondrocyte death^(^ ^75^ ^)^
				Delayed blood vessel invasion and formation of the primary and secondary ossification centers^(^ ^75^ ^)^
		Shortened forelimbs and hindlimbs^(^ ^75^ ^)^	Loss of HIF‐2α in limb bud mesenchyme caused modest transient delay in chondrocyte hypertrophy due to impaired differentiation of hypertrophic chondrocytes into late hypertrophic cells^(^ ^85^ ^)^	Shorter and developmentally delayed fetal bones via chondrocyte differentiation impairment and delayed terminal differentiation^(^ ^75^ ^)^
		Delayed chondrogenic differentiation and reduced *Col2a1* mRNA^(^ ^75^ ^)^	Dual *Hif1a; Hif2a* cKO phenocopied *Hif1a* cKO^(^ ^80^ ^)^	Dual *Vhl; Hif1a* cKO phenocopied *Hif1a* cKO^(^ ^75^ ^)^
				Dual *Vhl; Hif2a* cKO accelerated replacement of cartilage by bone resulting in complete disappearance of hypertrophic chondrocyte layer but no medullary cavity effect^(^ ^75^ ^)^
*Col2a1‐ER*	*Phd2* cKO ^(^ ^72^ ^)^	Accelerated osteoarthritis development post IL‐1β treatment in femoral head cartilage tissue via enhanced *Mmp13* expression^(^ ^81^ ^)^		No effect on cortical thickness in femora. Striking increase in trabecular bone volume and number in aged murine femora and vertebrae^(^ ^76^ ^)^
	Increased trabecular and cortical bone^(^ ^72^ ^)^			
	Increased bone formation in primary spongiosa and reduced resorption in secondary spongiosa^(^ ^72^ ^)^			No changes in calvaria morphology^(^ ^76^ ^)^
	No effect on white blood cell and red blood cell count or hematocrit^(^ ^72^ ^)^			Increased osteogenesis resulting in increased woven bone generation^(^ ^76^ ^)^
*Osx‐cre*	*Phd1 cKO* , *Phd2 cKO* , or *Phd3* cKO ^(^ ^71^ ^)^	No skeletal phenotype^(^ ^82^ ^)^	No skeletal phenotype^(^ ^82^ ^)^	High bone mass phenotype and heavily vascularized bone marrow microenvironment with dilated blood vessels^(^ ^77^ ^)^
	No skeletal phenotype nor any effect on mature myeloid and lymphoid blood lineages^(^ ^71^ ^)^			
	*Phd1; Phd2* cKO ^(^ ^71^ ^)^			
	Did not affect cortical bone thickness but significantly increased trabecular volume and number^(^ ^71^ ^)^			
	No effect on mature myeloid and lymphoid blood lineages^(^ ^71^ ^)^			
	No differences in microvascular density and blood vessel number^(^ ^71^ ^)^			
	*Phd2; Phd3* cKO ^(^ ^71^ ^)^			
	Did not affect cortical bone thickness but generated striking increase in trabecular volume and trabecular number independent of VEGF‐driven bone microvascular density^(^ ^71^ ^)^	Dual *Hif1a; Hif2a* cKO decreased trabecular bone volume fraction^(^ ^71^ ^)^		No effect on osteoclast number but impaired osteoclast function^(^ ^77^ ^)^
	No differences in microvascular density and blood vessel number^(^ ^71^ ^)^			
	Impaired primary spongiosa development with increased cartilage remnants in trabeculae of tibias^(^ ^71^ ^)^			
	No change in osteoblast number, mineral apposition rate, and bone formation rate but significantly increased *Opg* expression and reduction in trabecular and endosteal osteoclasts^(^ ^71^ ^)^			
	*Phd1; Phd3* cKO ^(^ ^71^ ^)^			
	No skeletal phenotype nor any effect on mature myeloid and lymphoid blood lineages			
	*Phd1; Phd1; Phd3* cKO ^(^ ^71^ ^)^			
	Excessive increase in trabeculae in metaphyseal and diaphyseal regions of long bones, decreased cortical thickness, and diminished bone marrow cellularity^(^ ^71^ ^)^			
*Col1a2‐iCre*	*Phd2 cKO* ^(^ ^73^ ^)^		*Hif2a* ^ *+/f* ^ *cKO*	
*Col1a1‐cre*	Decreased trabecular and cortical bone mass due to reduced bone formation rate; bone resorption unaffected^(^ ^73^ ^)^		Increased trabecular bone mass via enhancement of osteoblast differentiation and reduced osteoclast differentiation^(^ ^86^ ^)^	
	Appendicular skeleton affected; axial unaffected^(^ ^73^ ^)^		No cortical phenotype was observed^(^ ^86^ ^)^	
	Decreased in vitro osteoblast differentiation^(^ ^73^ ^)^			
*Bglap‐cre*		Decreased trabecular bone volume fraction, cortical cross‐sectional area, and vascularity^(^ ^78^, ^83^ ^)^	Moderate decreases in trabecular bone volume fraction and cross‐sectional area but maintained reduced vascular density^(^ ^78^, ^83^ ^)^	High bone mass^(^ ^78^ ^)^ with excessive trabeculae extended far into the diaphyseal bone shaft, associated with thinner, highly porous, trabecularized cortical bone but reduced skeletal growth^(^ ^77^ ^)^
		Dual *Hif1a;Vhl* cKO generated a high bone mass phenotype less severe than *Vhl* deletion alone^(^ ^78^ ^)^		Increased vessel volume^(^ ^77^, ^78^ ^)^
				Increased bone formation rate and Ob.N^(^ ^83^ ^)^ but no change in Ocl.N/BS or Opg^(^ ^78^ ^)^
				Appendicular skeleton affected; axial unaffected^(^ ^78^ ^)^
*Dmp1‐cre*	*Phd2* cKO ^(^ ^74^ ^)^	No effect on femoral cortical or trabecular phenotype^(^ ^79^ ^)^		High bone mass phenotype with increased trabecular and cortical bone^(^ ^79^ ^)^
	Increased bone mass via enhancement of bone remodeling in young mice and reducing bone resorption in aging mice^(^ ^74^ ^)^			No apparent changes in osteoclast activity^(^ ^79^ ^)^
				Decreased sclerostin, increased β‐catenin signaling^(^ ^79^ ^)^
	Decreased sclerostin expression via SIRT1 and enhanced skeletal angiogenic response^(^ ^74^ ^)^			*Vhl* cKO cortical phenotype required Wnt signaling, whereas trabecular phenotype independent of *Lrp5* and not resolved by sclerostin overexpression^(^ ^79^ ^)^
*Ctsk‐cre*		HIF‐1α knockdown had no effect on osteoclast differentiation but prevented the increase in osteoclast‐mediated bone resorption and glycolysis under hypoxia^(^ ^84^ ^)^	Oc/B.Pm and Oc.S/BS were significantly decreased, whereas Ob/B.Pm and Ob.S/BS were unaffected^(^ ^86^ ^)^	
		No effect on glucose uptake in basal normoxic conditions^(^ ^84^ ^)^	HIF‐2α siRNA knockdown moderately reduced the number of osteoclasts formed during osteoclastogenesis in vitro but did not affect osteoclast‐mediated bone resorption or glucose uptake^(^ ^84^ ^)^	

**Table 2:** Effect of HIF‐α Isoform‐Specific Accumulation on the Skeleton

Cre driver/gene	HIF‐1α cDR	HIF‐2α cDR
*Prx‐cre*		Increased trabecular bone microarchitecture but severe cortical bone thinning^(^ ^71^ ^)^
		Heterozygous overexpression does not affect BMSC commitment to osteogenic lineage in vitro^(^ ^80^ ^)^
		Heterozygous overexpression decreases osteoblast differentiation gene expression, increased osteoprotective *Opg* expression, and increased chrondrogenic differentiation via enhanced Sox9 expression in vivo^(^ ^88^ ^)^
*Osx‐cre*	No significant changes in cortical bone, nor trabecular bone volume fraction, trabecular number, or trabecular and endosteal N.Oc/BS^(^ ^71^ ^)^	Increased trabecular bone microarchitecture and diminished trabecular and cortical osteoclast number^(^ ^71^ ^)^
		No cortical bone phenotype; delayed chondrocyte hypertrophy at the growth plate. Decreased body weight and limb length^(^ ^71^ ^)^
*Bglap‐cre*		No effect on *Opg*; increased *Rankl* transcript and *Rankl:Opg* during calvarial preosteoblast differentiation^(^ ^86^ ^)^

### Structural and cellular composition of bone

The skeleton serves essential roles in support and protection of vital organs, maintaining mineral homeostasis, facilitating locomotion, and hematopoesis.^(^ ^63^ ^)^ Bone is composed of organic matrix (type 1 collagen and noncollagenous proteins) and inorganic salts called hydroxyapatite.^(^ ^64^, ^65^ ^)^ Collagen and noncollagenous proteins of the organic matrix form a scaffold where hydroxyapatite crystals are deposited between collagen fibers, which contributes to bone stiffness and resistance to compression that dictates its biomechanical integrity.^(^ ^66^ ^)^

There are two types of bone: cortical (compact) bone that forms the dense, protective outer layer of long bones, and trabecular (cancellous) bone that forms a lattice structure composed of struts and rods within the epiphyses/metaphases of long bones and within flat and irregular bones.^(^ ^64^ ^)^ Trabecular bone lattices efficiently redistribute mechanical loads imposed on the joints to the dense cortical bone that can better withstand high applied loads.^(^ ^64^ ^)^

Bone tissue is composed of four main cell types: bone lining cells, osteoblasts, osteocytes, and osteoclasts.^(^ ^63^ ^)^ Active remodeling sites harbor osteoblasts responsible for bone formation and/or osteoclasts responsible for bone resorption at the bone surface, whereas bone‐lining cells cover nonremodeling bone surfaces.^(^ ^67^ ^)^ Osteocytes are terminally differentiated osteoblasts that are embedded deep within the bony matrix and live on the order of decades.^(^ ^68^ ^)^ These long‐lived cells are hailed as the “master regulator of bone remodeling” because of their orchestrating of osteoblast and osteoclast activity via paracrine secretion of growth factors and cytokines^(^ ^69^ ^)^ and direct cell‐cell communication via gap junctions.^(^ ^70^ ^)^

Bone is a dynamic structure, continuously being formed and resorbed in a delicate balance to maintain appropriate bone mass and structural integrity after each remodeling cycle. This requires highly regulated cross‐talk between bone remodeling cells of the osteoblast and osteoclast lineages that is predominantly mediated by osteocytes. One such regulatory pathway, VHL/HIF signaling, is highly active in these cells, and its manipulation generates dramatic skeletal phenotypes.

### Effects of PHD and VHL manipulation in osteogenic cells

PHD and VHL are key regulatory proteins in the HIF pathway, whose roles in determining bone mass phenotypes are apparent. There are three PHD isoforms (PHD1, PHD2, PHD3) that serve as oxygen sensors to limit HIF signaling, and deletion of individual isoforms exerts varied impact on skeletal phenotype.^(^ ^65^ ^)^ Deletion of osteoprogenitor *Phd1* (*Osx‐cre*; *Phd1* ^*f/f*^), *Phd2* (*Osx‐cre*; *Phd2* ^*f/f*^), or *Phd3* (*Osx‐cre*; *Phd3* ^*f/f*^) did not affect skeletal phenotype.^(^ ^71^ ^)^ Dual deletion of *Phd1* and *Phd3* (*Osx‐cre*; *Phd1* ^*f/f*^ *; Phd3* ^*f/f*^) showed no differences in trabecular or cortical bone.^(^ ^71^ ^)^ In contrast, *Phd2* deletion in murine mesenchymal osteochondral progenitor cells (*Col2‐CreER* ^*T2*^; *Phd2* ^*f/f*^) causes high bone mass in both cortical and trabecular compartments involving increased bone formation and reduced resorption.^(^ ^72^ ^)^ Skeletal impact of *Phd* deletion was pronounced after combinatorial deletion of *Phd1* and *Phd2* or *Phd2* and *Phd3*. Each combination of *Phd1/Phd2* or *Phd2/Phd3* cKO in osteoprogenitors (*Osx‐cre*; *Phd1* ^*f/f*^ *; Phd2* ^*f/f*^ or *Osx‐cre*; *Phd2* ^*f/f*^ *; Phd3* ^*f/f*^) significantly increased trabecular microarchitecture without impacting cortical compartments; nor was there any impact on microvascular density or blood vessel number.^(^ ^71^ ^)^ *Osx‐cre*; *Phd2* ^*f/f*^ *; Phd3* ^*f/f*^ mice showed unique increases in *Opg* expression and reduced trabecular and endosteal osteoclast number and impaired spongiosa development characterized by increased cartilage remnants in tibial trabeculae.^(^ ^71^ ^)^ In osteoblast lineage cells, deletion of *Phd2* (*Col1a2‐iCre*; *Phd2* ^*f/f*^) decreased trabecular and cortical bone mass of long bones and reduced osteoblastic differentiation marker expression (*Osx*, *Bglap*, and *Bsp*), whereas bone resorption was unaffected.^(^ ^73^ ^)^ Osteocyte‐specific deletion of *Phd2* (*Dmp1‐cre; Phd2* ^*f/f*^) generated a significant increase in trabecular and cortical bone mass, wherein bone remodeling was enhanced in young mice and bone resorption was reduced in aging mice, ultimately increasing bone strength.^(^ ^74^ ^)^ *Phd2* is highly expressed in osteocytes compared with other osteogenic cells such as periosteum‐derived cells, trabecular osteoblasts, and bone marrow cells, and may confer the skeletal consequences of *Phd* deletion in progenitor cells.^(^ ^74^ ^)^

VHL is the master regulator of HIF activity by modulating its degradation. *Vhl* deletion in murine early limb‐bud mesenchymal cells (*Prx1‐cre; Vhl* ^*f/f*^) generated shortened, developmentally delayed fetal bones, delayed blood vessel invasion and bone marrow cavity development, impaired chondrocyte differentiation, and massive postnatal chondrocyte death.^(^ ^75^ ^)^ In mesenchymal osteochondral progenitor cells, conditional deletion of *Vhl* (*Col2‐CreER* ^*T2*^; *Vhl* ^*f/f*^) promotes formation of woven bone and striking increases in trabecular bone microarchitecture in aged (12‐month‐old) murine femora compared with *cre*‐negative controls, protecting them from age‐induced bone loss.^(^ ^76^ ^)^ *Col2‐CreER* ^*T2*^; *Vhl* ^*f/f*^ mice showed no cortical bone or calvarial phenotype.^(^ ^76^ ^)^ *Vhl* deletion in osteoprogenitors (*Osx‐cre; Vhl* ^*f/f*^) generated a high bone mass phenotype and dramatically increased vascularization with dilated blood vessels in the bone marrow microenvironment.^(^ ^77^ ^)^ Osteoblast‐specific V*hl*‐deficient mice (*Bglap‐cre; Vhl* ^*f/f*^) increased vessel volume and high bone mass phenotype with extensive trabeculae extended proximally into the femoral diaphysis and thinner, highly porous trabecularized cortical bone, yet reduced skeletal growth.^(^ ^77^, ^78^ ^)^ *Bglap‐cre; Vhl* ^*f/f*^ mice demonstrated increased bone formation rate (BFR) without affecting resorption.^(^ ^77^, ^78^ ^)^ Similarly, osteocytes lacking *Vhl* (*Dmp1‐cre; Vhl* ^*f/f*^)^(^ ^79^ ^)^ generated a high bone mass phenotype with augmented trabecular and cortical bone without changes in osteoclast activity.^(^ ^79^ ^)^ This phenotype was partially dependent upon Wnt/ß‐catenin signaling expression of the Wnt antagonist Sclerostin was reduced, with concomitant increases in activated ß‐catenin, in *Dmp1‐cre; Vhl* ^*f/f*^ mice. Compound *Vhl/Wnt* cKO mice revealed compartment‐specific impact on skeletal microarchitecture, wherein trabecular phenotype was intermediate between control and *Vhl* cKO mice, whereas cortical phenotype was indistinguishable from wild‐type mice.^(^ ^73^ ^)^

### Effects of HIF‐α isoform deletion in osteogenic cells

Because skeletal phenotypes resulting from PHD or VHL manipulation are the product of both HIF‐1α‐ and HIF‐2α‐mediated effects, single HIF‐α isoform manipulation is required to elucidate HIF‐α isoform‐specific functions in bone cells. *Hif1a* deletion in murine early limb‐bud mesenchymal cells (*Prx1‐cre; Hif1a* ^*f/f*^) delayed chondrogenic differentiation (reduced *Col2a1* expression), inducing massive chondrocyte death and severe limb abnormalities characterized by shortened forelimbs and hindlimbs.^(^ ^75^, ^80^ ^)^ Mice lacking *Hif1a* in murine mesenchymal osteochondral progenitor cells (*Col2‐CreER* ^*T2*^; *Hif1a* ^*f/f*^) treated with IL‐1ß to induce osteoarthritis showed accelerated osteoarthritis development in femoral head articular cartilage compared with IL‐ß‐treated control mice.^(^ ^81^ ^)^ This accelerated osteoarthritis phenotype was attributed to enhanced MMP13 expression, a catabolic enzyme for cartilage matrix.^(^ ^81^ ^)^ *Osx‐cre; Hif1a* ^*f*/f^ mice revealed no skeletal phenotype.^(^ ^82^ ^)^ Osteoblast‐specific *Hif1a*‐deficient mice (*Bglap‐cre; Hif1a* ^*f/f*^) presented decreased trabecular bone volume fraction, cortical cross‐sectional area, and vascularity,^(^ ^78^, ^83^ ^)^ whereas dual deletion of *Hif1a* and *Vhl* cKO (*Bglap‐cre; Hif1a* ^*f/f*^; *Vhl* ^*f/f*^) generated an intermediate phenotype compared with the high bone mass phenotype observed in the *Vhl* cKO (*Bglap‐cre; Vhl* ^*f/f*^).^(^ ^78^ ^)^ *Hif1a* deletion in osteocytes (*Dmp1‐cre; Hif1a* ^*f/f*^) had no effect on skeletal phenotype, and these mice were indistinguishable from *cre*‐negative controls.^(^ ^79^ ^)^ HIF‐1α knockdown in osteoclasts had no effect on osteoclast differentiation, but it prevented the increase in osteoclast‐mediated bone resorption and glycolysis under hypoxia; glucose uptake under normoxic conditions was unaffected.^(^ ^84^ ^)^

HIF‐1α manipulation in osteogenic cells is extensively considered yet passing consideration of HIF‐2α is common. Mice lacking *Hif2a* in early limb‐bud mesenchymal cells (*Prx1‐cre; Hif2a* ^*f/f*^) revealed a high bone mass phenotype with increased trabecular and cortical bone without changes in osteoclast number.^(^ ^80^ ^)^ In a separate study, these mice had a modest, transient delay in chondrocyte hypertrophy due to impaired differentiation of hypertrophic chondrocytes to late hypertrophic cells but no significant growth deformities.^(^ ^85^ ^)^ *Hif1a*‐deficient mice (*Prx1‐cre; Hif1a* ^*f/f*^) had severe limb abnormalities, delayed chondrogenic differentiation, and massive chondrocyte death. Dual deletion of *Hif1a* and *Hif2a* (*Prx1‐cre; Hif1a* ^*f/f*^ *; Hif2a* ^*f/f*^) phenocopied *Hif1a* cKO mouse phenotype.^(^ ^80^ ^)^ In contrast, dual deletion of *Hif2a* and *Vhl* (*Prx1‐cre; Hif2a* ^*f/f*^ *; Vhl* ^*f/f*^) accelerated replacement of cartilage by bone, completely diminishing the hypertrophic chondrocyte layer but having no effect on the medullary cavity.^(^ ^75^ ^)^ Osteoprogenitor‐specific deletion of *Hif2a* (*Osx‐cre; Hif2a* ^*f*/f^) produced a phenotype indistinguishable from *cre*‐negative controls; however, dual deletion of *Hif1a* and *Hif2a* (*Osx‐cre; Hif1a* ^*f*/f^ *; Hif2a* ^*f*/f^) decreased trabecular bone volume and did not affect cortical bone phenotype.^(^ ^71^ ^)^ Heterozygous *Hif2a* deletion in osteoblasts (*Col1a1‐cre; Hif2a* ^*+/f*^) increased trabecular bone microarchitecture via enhanced osteoblast differentiation and reduced osteoclastogenesis; no cortical phenotype was observed.^(^ ^86^ ^)^ Osteoblastic *Hif2a* (*Bglap‐cre; Hif2a* ^*f/f*^) deletion reduced trabecular bone volume fraction and cross‐sectional area despite maintaining reduced vascular density.^(^ ^78^, ^83^ ^)^ In vitro studies revealed that calvarial osteoblast precursor cells undergoing osteoblastic differentiation had increased expression of osteocalcin (OCN) and RUNX2, two known osteoblast differentiation markers, and a concomitant decrease in HIF‐2α expression, wherein HIF‐2α blocks osteoblastic differentiation via twist family BHLH transcription factor 2 (*Twist2*).^(^ ^86^ ^)^ *Twist2* siRNA‐mediated knockdown prevented downregulation of *Ocn* and *Runx2* by HIF‐2α in vitro, whereas immunohistochemical in vivo studies revealed association between HIF‐2α and TWIST2.^(^ ^86^ ^)^ Osteoclast‐specific deletion of *Hif2a* (*Ctsk‐cre; Hif2a* ^*f/f*^) significantly reduced osteoclast activity, whereas osteoblast differentiation and activity were unaffected.^(^ ^86^ ^)^ In a separate study, siRNA‐mediated osteoclastic HIF‐2α knockdown moderately reduced the number of osteoclasts formed during osteoclastogenesis in vitro; however, osteoclast‐mediated bone resorption and glucose uptake remained unaffected under normoxic conditions.^(^ ^84^ ^)^ HIF‐1α siRNA had no effect on osteoclastic differentiation, inhibition of hypoxic bone resorption, or glycolysis.^(^ ^84^ ^)^

### Effects of degradation‐resistant HIFs in osteogenic cells

To elucidate the roles of specific HIF‐α isoforms in hypoxia, each HIF‐α isoform must be evaluated in conditions where they are constitutively stable/their levels are constitutively elevated. A gain‐of‐function mutation is generated via nucleotide substitutions at two proline residues (Pro‐405A; Pro‐531A), resulting in the replacement of proline by alanine residues (termed dPA; delta proline to alanine substitution) at the HIF‐α hydroxylation site, preventing oxygen‐dependent prolyl hydroxylation and subsequent VHL recognition and degradation. HIF‐1α dPA^f/f^ and HIF‐2α dPA^f/f^ mice containing mutant human cDNA encoding HIF‐1α dPA and HIF‐2α dPA protein are knocked into the ROSA26 locus preceded by a lox‐stop‐lox (LSL) cassette.^(^ ^87^ ^)^ Upon Cre‐mediated recombination, the STOP sequence is removed, allowing for expression of the mutated coding sequence. LSL‐HIF‐1α^DPA^ mice will henceforth be called HIF‐1α conditional‐degradation resistant (HIF‐1α cDR), and LSL‐HIF‐2α^DPA^ mice will be called HIF‐2α conditional‐degradation resistant (HIF‐2α cDR).

HIF‐2α cDR expression in mesenchymal osteoprogenitors (*Prx1‐cre*; HIF‐2α cDR^+/f^) markedly increased *Vegf* and *Opg* expression; however, *Runx2* and *Col1A1* (known osteogenic cell commitment marker) expression remained unaffected, indicating that BMSC‐specific HIF‐2α cDR does not affect BMSC commitment to osteogenic lineage in vitro.^(^ ^80^ ^)^ These mice also showed significant decreases in key osteoblast differentiation genes (*Sp7*, *Ibsp*, *Alp1*), increased osteoprotective *Opg* expression, and enhanced expression of the chondrogenic and anti‐osteogenic *Sox9* in vivo,^(^ ^88^ ^)^ suggesting that HIF‐2α impairs differentiation of osteoprogenitors into osteoblasts. These mice had significant decreases in osteoblast number and activity, generating a severe cortical bone thinning phenotype; however, these mice exhibited significantly increased trabecular bone due to increased *Opg* expression in the trabecular compartment.^(^ ^71^ ^)^ Similarly, osteoprogenitor‐specific HIF‐2α cDR (*Osx‐cre*; HIF‐2α cDR) also showed increases in trabecular bone and diminished trabecular and endosteal osteoclasts via increased *Opg* expression.^(^ ^71^ ^)^ In contrast, *Osx‐cre*; HIF‐2α cDR mice did not show a cortical bone phenotype but did show significantly delayed chondrocyte hypertrophy at the growth plate and significant decreases in body weight and limb length.^(^ ^71^ ^)^ Osteoprogenitor‐specific HIF‐1α cDR (*Osx‐cre*; HIF‐1α cDR) did not affect skeletal phenotype.^(^ ^71^ ^)^ Osteoblast‐specific HIF‐2α cDR (*Bglap‐cre*; HIF‐2α cDR) had no effect on *Opg* expression; however, *Rankl* transcript levels increased (increasing *Rankl:Opg* ratio) during preosteoblast differentiation.^(^ ^86^ ^)^ These results suggest that HIF‐2α directly inhibits osteoclastogenesis via decoy OPG expression in osteoprogenitors and osteoblasts.^(^ ^71^, ^80^ ^)^ Our studies show that mice expressing HIF‐2α cDR in osteocytes (*Dmp1‐cre*; HIF‐2α cDR) had rampant cortical thickening with increased porosity and increased trabecular bone volume and trabecular number.^(^ ^89^ ^)^ A similar phenotype has been observed in female mice lacking the SOCS3 cytokine signaling suppressor, exhibiting increased trabecular bone and poor corticalization.^(^ ^90^ ^)^ The changes in cortical and trabecular parameters of the HIF‐2α cDR mouse resemble what was reported in the *Dmp1cre*; *Vhl* ^*f/f*^ cKO mouse; however, the HIF‐2α cDR phenotype only partially recapitulates the *Vhl* cKO HBM phenotype.^(^ ^89^ ^)^ The *Dmp1‐cre*; HIF‐2α cDR mouse, in contrast to *Dmp1cre*; *Vhl* ^*f/f*^ cKO mouse, also demonstrated osteogenic stromal expansion in the distal femoral bone marrow compartment that occurred at the expense of hematopoietic lineages and significant increases in osteoclast activity within the distal femoral metaphyseal region.^(^ ^89^ ^)^ These data suggest that osteocytic HIF‐2α accumulation upregulates osteoclastogenesis, resulting in increased bone turnover, increased osteoblastic differentiation, and trabecular bone formation.

## HIF‐2α Signaling in Skeletal Development and Disease

Clearly, the VHL/HIF pathway has significant effects on the skeleton. HIF‐1α‐focused research makes up the majority of what is currently known about the hypoxia‐inducible signaling pathway and its downstream effects. In the following sections, we will focus on the lesser studied HIF‐2α and its role in different tissues and pathophysiology.

### Endochondral ossification and cartilage development

Hypoxia is intimately associated with endochondral ossification, a process in which mesenchymal stem cells condense and differentiate into chondrocytes, forming a cartilaginous blueprint for subsequent bone development. Proliferating chondrocytes undergo hypertrophy, secrete type X collagen (COL10A1), and calcify surrounding cartilage, creating a locally hypoxic environment.^(^ ^91^ ^)^ Hypertrophic chondrocytes express high levels of VEGF that induce vascular invasion, supplying nutrients and osteoprogenitors to support avascular cartilage replacement with vascularized bone wherein osteoclasts resorb calcified cartilage and osteoblasts form new bone.^(^ ^92^ ^)^

HIF‐2α expression is localized in hypertrophic growth plate chondrocytes and articular chondrocytes in the superficial zone.^(^ ^93^ ^)^ HIF‐2α expression increased with chondrocyte maturation in differentiating murine ATDC5 cells; expression of vascular endothelial growth factor, placental growth factor, and glucose transporter 1, known hypoxia‐responsive genes, paralleled this increase in HIF‐2α,^(^ ^93^ ^)^ suggesting that HIF‐2α may play a role in bone development by regulating vascularization and promoting anaerobic metabolism in chondrocytes. HIF‐2α is a potent transactivator of COL10A1, as it contains an HRE in the *COL10A1* promoter that binds HIF‐2α.^(^ ^91^ ^)^ Adenoviral HIF‐2α overexpression in murine chondrogenic ATDC5 cells increased expression of endochondral ossification markers: *Col10a1*, *Mmp13*, and *Vegfa*.^(^ ^91^ ^)^ This functional association was further supported with in vivo studies deleting *Mmp13* (*ß‐actin‐cre; Mmp13* ^*f/f*^) or *Vegfa* (*Prx1*‐*cre*; *Vegfa* ^*f/f*^), which phenocopied mice with heterozygous *Hif2a* deletion.^(^ ^91^, ^94^, ^95^ ^)^ Mice with heterozygous *Hif2a* deletion showed a mild dwarfism phenotype with defects in the central steps of endochondral ossification but no major organ development abnormalities.^(^ ^91^ ^)^ These mice exhibited a decrease in relative hypertrophic zone to the total limb length, suggesting that all key endochondral ossification steps (chondrocyte hypertrophy, matrix degradation, and vascularization) were impaired.^(^ ^91^ ^)^ In contrast, mice with complete loss of *Hif2a* were extremely small and died during early embryonic stage.^(^ ^91^ ^)^ Thus, HIF‐2α is essential for endochondral ossification of chondrocytes and embryonic skeletal growth in mice.^(^ ^91^ ^)^

### Aging bone

Senescence is characterized by gradual deterioration of structure and function with age. At the cellular level, it contributes to age‐related tissue dysfunction like irreversibly arrested cell growth and widespread changes in chromatin organization and gene expression.^(^ ^96^ ^)^ A senescent state is favorable for a damaged cell, by limiting damage propagation to cell progeny and terminating potential malignancy.^(^ ^97^ ^)^ Senescent cells induce changes in neighboring cells via secretion of pro‐inflammatory and matrix‐degrading factors that create a toxic microenvironment called the senescence‐associated secretory phenotype (SASP).^(^ ^98^ ^)^ A key characteristic of cellular senescence is cell cycle arrest resulting from the action of cyclin‐dependent kinase inhibitor p16 (*Cdkn2a*) and p21 (*Cdkn1a*).^(^ ^99^ ^)^

Senescent p16‐expressing cells induce tissue dysregulation that can significantly shorten life span. Clearance of murine p16‐positive senescent cells even in mild proportions (~30%) via drug‐induced (AP20187) activation of *INK‐ATTAC*—a transgene expressed in p16‐positive/senescent cells that when drug‐induced results in targeted apoptosis—beginning at 12 months of age until the end of life, increased life span in male and female mice from two genetic backgrounds and prevented senescence‐induced tissue dysregulation.^(^ ^100^ ^)^ A separate study focusing on the skeletal effects of p16‐clearance used the drug‐induced (AP20187) *INK‐ATTAC* transgene treatment starting at 12 months of age (young cohort) and 20 months of age (old cohort) for 4 months.^(^ ^101^ ^)^ In bone, p16 mRNA expression decreased by 48%, respectively, and p16‐positive osteocytes were reduced by 46%, respectively, compared with vehicle control.^(^ ^101^ ^)^ AP20187 restored trabecular bone volume in spine and femora in old mice via reduced resorption upon clearance of senescent cells, while maintaining bone formation rate compared with aged vehicle control.^(^ ^101^ ^)^ In vivo SASP suppression in 22‐month‐old mice with ruxolitinib treatment, a JAK1/2 inhibitor (JAKi), prevented age‐related bone loss in spine and femur and enhanced femoral bone strength.^(^ ^101^ ^)^ This occurred via reduction of the senescent cell population, reducing osteoclast number/bone resorption yet maintaining osteoblast number/bone formation, suggesting a functional uncoupling between osteoclasts and osteoblasts.^(^ ^101^ ^)^ Taken together, in vivo reduction in senescent cell burden prevented age‐related reductions in bone mass.

Osteocytes from aged mice express senescent markers at a significantly higher level compared with other cells in the bone microenvironment, namely osteoblast progenitors, osteoblasts, and hematopoietic lineages.^(^ ^102^ ^)^ Although p16 and p21 are senescence markers, how they functionally contribute to SASP requires greater elucidation, especially in the context of varied expression across cells from aged animals. Senescent osteocytes in bones of aged mice express elevated levels of p16 only,^(^ ^102^ ^)^ senescent osteoblast progenitors express elevated levels of p21 only,^(^ ^103^ ^)^ whereas osteoclasts express elevated levels of both p16 and p21 during osteoclast differentiation.^(^ ^104^ ^)^ *Hif2a* mRNA expression significantly increases with age, concomitant with age‐induced increases in p16 and p21 in whole femur homogenates.^(^ ^104^ ^)^ This association was verified with ChIP experiments wherein HIF‐2α binds HREs in the promoters of p16 and p21 in aging osteoclasts, directly enhancing their transcription.^(^ ^105^ ^)^ Furthermore, osteoclast‐specific *Hif2a* deletion (*Ctsk‐cre; Hif2af/f*) significantly reduced p16 and p21 expression. Heterozygous *Hif2a* deletion reversed age‐induced osteoporotic bone loss in aged (12‐month‐old mice), where trabecular microarchitecture was significantly increased, and cortical bone was unaffected when compared with age‐matched controls.^(^ ^105^ ^)^ This suggests that HIF‐2α functioning as a senescence‐gene activator negatively affects skeletal phenotype.

Senescent p21‐positive osteoblasts from aged mice (12‐month‐old) exhibit reduced *Bglap* and *Runx2* expression and impaired osteoblast differentiation, with concomitant increases in *Hif2a* expression.^(^ ^105^ ^)^ Induction of doxorubicin‐mediated osteoblast senescence during osteoblast differentiation significantly increased *Hif2a* expression while significantly reducing osteoblast differentiation marker gene expression (*Bglap* and *Runx2*).^(^ ^105^ ^)^ In a separate study, the HIF‐2α target *Twist2* inhibited *Runx2* expression during early osteogenic differentiation and reduced *Bglap* expression; therefore, senescence‐associated upregulation of HIF‐2α may dysregulate osteoblast function in part by *Twist2*.^(^ ^86^ ^)^ Furthermore, treatment of induced‐senescent osteoblasts with a HIF‐2α inhibitor resulted in recovery of osteoblast function in vitro.^(^ ^105^ ^)^ The in vivo role of osteoblastic HIF‐2α as a negative regulator of skeletal phenotype was elucidated in mice with osteoblast‐specific depletion of HIF‐2α (*Col1a1‐cre; Hif2a* ^*f/f*^), where trabecular microarchitecture and bone mass were preserved with aging, preventing age‐dependent osteoporotic bone loss.^(^ ^105^ ^)^

Studies investigating the role of HIF‐2α in age‐induced senescent osteocytes are remarkably absent. Because osteocytes are long‐lived cells that make up more than 95% of all bone cells,^(^ ^106^ ^)^ their senescence‐associated contribution to skeletal phenotype is significant. Senescent osteocytes express several age‐related SASP factors like NF‐κB,^(^ ^107^ ^)^ matrix metalloproteinases (MMPs), interleukins, and inflammatory cytokines^(^ ^98^ ^)^ that can influence the secretory profile of healthy neighboring aged and young osteocytes.^(^ ^108^ ^)^ Osteocytes also express HIF‐2α, but an association between HIF‐2α and expression of senescence genes in these cells has not yet been investigated.

### Osteoporosis

Postmenopausal estrogen deficiency and advanced age are associated with dysregulated bone remodeling that is characterized by unbalanced osteoblast‐mediated bone formation and osteoclast‐mediated bone resorption.^(^ ^109^, ^110^ ^)^ This imbalance skews bone remodeling toward osteoclastic bone resorption, decreasing bone mass, impairing bone healing, and increasing risk for osteoporotic fracture.^(^ ^109^, ^110^ ^)^ Estrogen stimulates osteoblastic expression of osteoprotective osteoprotegerin (OPG), a decoy protein that sequesters the osteoclast activator receptor activator of NF‐κB ligand (RANKL), inhibiting osteoclast differentiation and reducing bone resorption.^(^ ^111^ ^)^ Therefore, reduced estrogen is associated with reduced OPG, increased osteoclast differentiation, and enhanced bone resorption. In addition, estrogen deficiency affects the osteogenic arm of remodeling by reducing the life span and function of osteoblasts.^(^ ^112^, ^113^ ^)^ With aging, adipogenic differentiation of bone marrow mesenchymal cells (BMMCs) preferentially occur, depleting the available BMMC population and reducing osteogenic lineage differentiation.^(^ ^114^ ^)^

Estrogen deficiency–induced increases in osteoclastic bone resorption and reduced osteoblastic bone formation may involve HIF signaling. Ovariectomy (OVX)‐induced estrogen deficiency increases both HIF‐1α^(^ ^115^ ^)^ and HIF‐2α^(^ ^86^ ^)^ levels in osteoclasts but reduces osteoblastic HIF‐1α and HIF‐2α expression.^(^ ^116^ ^)^ HIF‐1α expression was also increased in murine osteoclasts after orchidectomy.^(^ ^117^ ^)^ HIF‐α isoform stabilization induced by osteoblast‐specific *Vhl* deletion (*Bglap‐cre; Vhl* ^*f/f*^) or pharmacologic manipulation (dimethylyoxaglycine) prevents the bone loss and reduced vascularity observed in OVX mice.^(^ ^116^, ^118^ ^)^ Deletion of HIF‐1α^(^ ^115^ ^)^ or HIF‐2α^(^ ^86^ ^)^ in osteoclasts (*Ctsk‐cre*) also abrogates OVX‐induced bone loss in mice. These data suggest HIF signaling is differentially regulated by estrogen in osteoblasts and osteoclasts with osteoclastic HIF signaling enhanced in estrogen deficiency, promoting bone resorption.

It should be noted that deletion of HIF‐2α in osteoblasts (*Col1a1‐cre; Hif2a* ^*f/f*^) also abrogates OVX‐induced bone loss in mice.^(^ ^86^ ^)^ This appears counterintuitive given the bone‐protective effects of osteoblast‐specific *Vhl* deletion, which should stabilize both HIF‐1α and HIF‐2α. However, mice with deletion of HIF‐2α in osteoblasts (*Col1a1‐cre; Hif2a* ^*f/f*^) were shown to have fewer osteoclasts and reduced bone resorption coupled with an increased bone volume.^(^ ^86^ ^)^ In vitro experiments demonstrated that osteoblasts deficient in HIF‐2α produced less RANKL and had impaired ability to support osteoclastogenesis in cocultures with osteoclastic precursors.^(^ ^86^ ^)^ Therefore, the osteoprotective effect of osteoblast HIF‐2α deletion in OVX mice was likely the result of impaired osteoblast‐mediated osteoclastogenesis.

### Osteoarthritis

Osteoarthritis (OA) is an acute or chronic joint inflammatory disease associated with joint damage and pain, and its acceleration is associated with HIF‐2α signaling. In a murine model of osteoarthritis, knee joint instability induced progressive cartilage degradation that was associated with increased HIF‐2α expression in chondrocytes.^(^ ^91^ ^)^ Global heterozygous deletion of *Hif2a* suppressed the development of osteoarthritis in the same surgical model.^(^ ^91^ ^)^ In a similar study, mice with heterozygous global deletion of *Hif2a* were resistant to development of osteoarthritis induced by collagenase injection or destabilization of the medial meniscus.^(^ ^119^ ^)^ HIF‐2α has also been detected in osteoarthritic human knee samples with increased expression levels correlated with disease progression, followed by decreased expression at the most severe stage.^(^ ^91^, ^119^ ^)^ HIF‐2α induces chondrocyte expression of a number of genes involved in matrix catabolism, including matrix metalloproteinase family members, aggrecanase‐1, nitric oxide synthase‐2, and prostaglandin‐endoperoxide synthase.^(^ ^119^ ^)^ Thus, it has been proposed that HIF‐2α may play a regulatory role in the development of osteoarthritis by regulating expression of these catabolic factors.

### Osteosarcoma

Osteosarcoma, or osteogenic sarcoma, is the most common type of malignant bone tumor in children and adults.^(^ ^120^ ^)^ Osteosarcomas house malignant osteoblasts that produce osteoid matrix and/or immature bone and thrive in a hypoxic environment.^(^ ^121^, ^122^ ^)^ Rapid growth of solid tumors depletes the available nutrient and blood supply, generating a hypoxic environment where HIF expression increases. Although HIF activation is well described in cancer and HIF‐1 expression is associated with poor outcomes in osteosarcoma,^(^ ^123^ ^)^ there are limited studies on the role of HIF‐2α. However, HIF‐2α expression has been detected in human osteosarcoma tissue with expression levels correlated with osteosarcoma stage.^(^ ^124^ ^)^

In most solid tumors, HIFs promote tumor progression and metastasis by increasing expression of genes associated with angiogenesis, proliferation, cell migration and invasion, extracellular matrix remodeling, and cell survival.^(^ ^125^, ^126^, ^127^ ^)^ Hypoxia may also reduce apoptosis and promote autophagy of tumor cells, increasing their resistance to radiation or chemotherapy treatment.^(^ ^128^, ^129^ ^)^ In in vitro studies, siRNA‐mediated knockdown of HIF‐2α in the MG‐63 osteosarcoma cell line reduced cell proliferation and increased apoptosis, likely through the MAPK‐p38 signaling pathway.^(^ ^130^ ^)^ Although HIF‐1α stabilization is implicated in osteosarcoma cell resistance to chemotherapeutic agents,^(^ ^131^, ^132^ ^)^ the role of HIF‐2α remains to be investigated. Elevated HIF‐2α levels are associated with poor prognosis in patients with neuroblastoma and cervical, kidney, bladder, breast, lung, and colorectal cancer.^(^ ^128^, ^133^, ^134^, ^135^, ^136^, ^137^, ^138^ ^)^ In ovarian, lung, brain, and kidney cancer cells, HIF‐2α upregulates the expression of CD70, a key cancer‐related antigen that enhances anchorage‐dependent growth in cancer cells, by inhibiting its methylation by DNA methyltransferase 1 (DNMT1), promoting aggressive cancer cell phenotypes.^(^ ^134^ ^)^ HIF‐2α inhibitors like the FDA‐approved Welireg, which blocks dimerization of HIF‐2α with HIF‐β,^(^ ^139^, ^140^ ^)^ diminishes HIF pathway activation and can prevent further tumor progression.^(^ ^141^ ^)^

HIF2‐α is expressed at high levels in tumor‐associated macrophages (TAM),^(^ ^142^ ^)^ and high TAM HIF‐2α levels are associated with higher‐grade tumors.^(^ ^143^ ^)^ HIF‐2α is expressed in hypoxic macrophages and promotes macrophage migration.^(^ ^144^ ^)^ TAMs create an immunosuppressive tumor microenvironment via cytokine, chemokine, and growth factor secretion.^(^ ^145^ ^)^ HIF‐1α and HIF‐2α have independent and opposite effects on tumor growth and spread in human and cultured murine macrophages.^(^ ^146^ ^)^ Deletion of *Hif2a* (but not *Hif1a*) in mouse macrophages significantly inhibited expression of macrophage colony‐stimulating factor 1 receptor (M‐CSFR) and chemokine receptor (CXCR4), reducing macrophage migration into tumors, delaying tumor progression, and reducing tumor burden.^(^ ^144^ ^)^ Although infiltration of macrophages into osteosarcoma has been described, the relationship between highly variable macrophage phenotypes and clinical prognosis is less clear.^(^ ^147^ ^)^ Taken together, these studies suggest that HIF‐2α may represent a useful prognostic marker in osteosarcoma and a novel therapeutic target, but further studies are required to fully understand its multifaceted roles.

### Regeneration and fracture healing

Disruption of the vasculature during bone fracture creates a localized hypoxic environment.^(^ ^148^, ^149^ ^)^ This serves as a powerful stimulus for the initiation of tissue regeneration, which requires progenitor cell invasion and differentiation, angiogenesis, and tissue formation.^(^ ^150^ ^)^ In a murine closed femoral fracture model, elevated transcripts for *Hif1a* and target gene *Vegf* were observed at all time points after fracture,^(^ ^151^ ^)^ with peak *Hif1a* transcript and protein expression observed 10 days post‐fracture. HIF‐1α and VEGF were observed in both proliferating chondrocytes and active osteoblasts, indicating participation of these proteins in endochondral bone formation. Similarly, both hypoxia and HIF‐1α expression were detected in regenerating bone in a murine model of distraction osteogenesis.^(^ ^152^ ^)^ Genetic or pharmacologic activation of HIF signaling via deletion of *Vhl* in osteoblasts (*Bglap‐cre; Vhl* ^*f/f*^) or desferrioxamine (DFO) treatment increased angiogenesis and bone regeneration in the distraction gap; conversely, HIF‐1α deletion in osteoblasts (*Bglap‐cre; Hif1a* ^*f/f*^) reduced bone regeneration.^(^ ^152^ ^)^ Pharmacological activation of HIF signaling (DFO and dimethyloxalylglycine) in a murine femur fracture model increased both callus size and vascularity,^(^ ^153^ ^)^ whereas DFO promoted bone bridging in a murine segmental defect.^(^ ^154^ ^)^ Taken together, these data strongly support a role for HIF signaling in bone regeneration but provide no insight into a specific role for HIF‐2α in this process.

## Summary and Conclusions

HIF‐2α has remained significantly understudied in skeletal tissues, where the phenotypic effects of hypoxia‐sensing pathway manipulation have typically been attributed solely to the activity of HIF‐1α. Future studies investigating the specific role of HIF‐2α are necessary and required for a holistic understanding of hypoxia signaling in the skeleton and in skeletal disease.

## Author Contributions

**Sarah V Mendoza:** Writing – original draft; writing – review and editing. **Damian Genetos:** Conceptualization; writing – review and editing. **Clare E. Yellowley:** Conceptualization; writing – review and editing.

## Disclosures

All authors declare that they have no relevant or material financial interests that relate to the research described in this article.

### Peer Review

The peer review history for this article is available at https://publons.com/publon/10.1002/jbm4.10733.
